# Looking Forward

**Published:** 1890-04

**Authors:** S. H. Preston


// HALL'S
### JOURNAL OF HEALTH

TRUTH DEMANDS NO SACRIFICE; ERROR CAN MAKE NONE.

Vol. 37. APRIL, 1890. No. 4.

### LOOKING FORWARD.

BY S. H. PRESTON.

No. 7.

JOSH BILLINGS believed there was a class of men who were not of the least use in this life. They were those that loafed around cider mills and village groceries, and did nothing but chew plug tobacco for a living. He believed thus till the cholera broke out. Then he discovered for what providence designed them. They helped make up the mortuary list. They counted when the cholera came.

Numbers do not necessarily add to the strength or stability of a nation. Population alone does not give a government greatness or prosperity. We could put an army of 100,000 cripples in the field, but one squad of such soldiers as Sherman marched to the sea would scatter it.

The more populous our poorhouses and penitentiaries, our asylums and city slums, the more is our great growth of population to be regretted rather than encouraged. To be sure all serve to swell the census, but we could spare enough to settle a State and be the better for it. It is only the intelligent, industrious, self-supporting citizens that count for the country's good.

To receive all the foreign refuse that comes to Castle Garden, all the slops of Europe poured into our ports, to take in trash that is not for our national nourishment and which cannot be assimilated into our social system, is like a glutton gulping down all before him regardless of his stomach. This general gobbling in of old-world garbage may some day give us governmental gripe.

Every pauper, every non-producer, every unemployed laborer, is a daily detriment to the Republic. They consume something and contribute nothing. Society is a joint stock concern. Those who put in nothing and take out something, those who do not replace their living, however little, are swindlers. They live like leeches on the labor of others. Those who do not produce what will pay their way are a clog on the people's progress.

Our Republic is a federal family—a family of States founded on the fireside families of the land. Therefore its chief concern should be the welfare of all the families it represents. It cannot afford to let any suffer, since the least cannot suffer without affecting the foundation of the fabric. That nation is the most fortunate and will flourish fastest which has the greatest number of frugal, full fed, felicitous families.

It is the popular impression that the family is something too private and sacred for any State supervision whatsoever. But in our great representative government, which is peculiarly one "of the people, by the people, and for the people," it becomes as much the business of the several States to attend to the condition of their citizens as it does of the citizens to see to the affairs of state. Because, to maintain good government, there must needs be a majority of good and useful citizens.

The prosperity of the Republic depends upon the personal prosperity of the people. So each of the States is allowed to legislate what will best promote the interests of its own inhabitants. Thus New York (selecting that State as an example) paternally provides for the general instruction of its children, and the preservation of the health and morals of its population. It has established by statute a system of free common schools. It interferes in family affairs so far as to see that every child has an education. It deems the instruction of the young of too much importance to be intrusted to parents themselves.

Also the matter of health. The State sees that every child is properly vaccinated before it enters the public school. And if a child be neglected by its natural guardians or be cruelly treated by them, the State will deprive them of its custody and provide for it in one of its public institutions. So that under certain circumstances the State dictates the duty of parents to their children, and even assumes control of them itself.

The State has societies chartered for the express purpose of looking after its prospective citizens. It can send its officers into the nursery to rescue the ill-cared for babe from its unnatural mother. It has other societies to look after the morals of the community at large, to protect the public from literary impurity and lottery imposture. It proscribes prints we should not sell, pictures we should not see, and devices of chance we should avoid. It has Boards of Health to see to the sanitary condition of our dwellings. And when we are sick it cannot trust us to choose our own physician. We might make a mistake, and our health is of too much importance to be exposed to individual indiscretion. So it makes it a misdemeanor for any but its own duly diplomatized doctors to meddle with medicine. Now all this is quite an interference in family affairs.

Considering that one family (the Jukes) cost New York $1,200,000; that such families are continually becoming charges upon the charities of the State; that it is the poor and improvident, the irresponsible and depraved who propagate the fastest; that families go right on increasing their numbers till they become burdens upon the public bounty; that among many families the birth of one more member will reduce them to destitution demanding charitable relief;—in the face of such facts it is not only right but required that the State interfere in family affairs, but to the end of restricting this rabbit-like reproduction of an undesirable population instead of promoting it. Better prevent parents from breeding paupers than punish them for preventing doing so themselves.

Parents should not be permitted to propagate children they cannot care for themselves, and the State has clearly a right to see that beings are not born that will become a public charge. It already exercises such a right under its bastardy acts. The putative father of a child that may become a charge of charity (or whose mother may), must furnish bonds for its support. If he does not, the State puts him in prison and sets him to work to satisfy the claim it has against him. The State says to an unmarried father, "you support that child; or you shall be shut up and made to work for it."

The sole concern of the State is that of support. Then what matters it whether the father be married or not? Does it not cost the State as much to care for the child of a married as of an unmarried man? The question is not as to the moral or legal right to beget children, but the privilege of propagating them at the public expense. The marriage license does not confer a right to raise charges upon State charity. A man cannot keep a dog without a tax license, but he can fill State institutions with his young ones *ad libitum*. It should signify naught to the State whether a pauper be the legitimate or illegitimate offspring of indigence. In either case it costs the same to keep him.

There is no way to prevent pauperism but to stop the production of paupers, from whatever source. And the State has a right to do this, and should do it as a duty to its thrifty, tax-paying citizens. It is as much its province to see that no family increases beyond the ability of the father to support it, as it is to see that children are sent to school and vaccinated. It only needs to make a general application of the laws now confined to cases of bastardy—to oblige all fathers, without distinction of marriage, to give bonds for the support of such of their children as may become a public charge.

We have stopped importing paupers. We do not now allow immigrants to land who are likely to become subjects of State support. The steamship company that brings them must carry them right back. But while we are sending back one foreign pauper New York furnishes five that are native born. And as long as the State provides for them and takes no steps to stop the supply, pauperism will grow far faster than population, as statistics show it has for the last half century.

But aside from the sole consideration of support, small families are of more service to the State. The fewer the children the better can be their care and education and culture, the better will be their opportunity to become worthy and useful citizens.

The country that has the smallest sized families contains the best conditions of prosperity and advancement. In illustration of this, contrast France, where families average from one to three in the different departments, with Ireland, where families average from five to seven. The former, one of the foremost, most enlightened and progressive of European powers, the latter but a by-word for beggary and degradation.

When Madame De Stael asked Napoleon what France most needed, he answered "Mothers." That was when mothers were wanted merely as multipliers of human material for wholesale murder, as breeders of butchers for the battle fields of Bonaparte. But mothers are no longer required solely as reproducers of the race, as the means of swelling the census. We want no more mothers to raise recruits for our reformatories or resorts of charity. Our States now only need mothers of well born, welcome children, whom they can comfortably care for and rear to become good and respected citizens.

